# Birt-Hogg-Dubé syndrome: case report and brief review of the literature

**DOI:** 10.1016/j.radcr.2021.10.052

**Published:** 2021-11-18

**Authors:** Dhairya A Lakhani, Lana Winkler, Mark Lisle

**Affiliations:** Department of Radiology, West Virginia University, Morgantown, WV 26506

**Keywords:** BHDS, Birt-Hogg-Dubésyndrome, Birt-Hogg-Dubé syndrome, BHDS, cystic lung disease

## Abstract

Birt-Hogg-Dubé syndrome (BHDS) is a rare autosomal-dominant multiorgan systemic disorder manifesting as cutaneous fibrofolliculomas, lung cysts with or without spontaneous pneumothorax, and renal tumors. It results from mutation of the gene located on the short arm of chromosome 17 (17p11.2). The gene codes for the protein folliculin, which is believed to be an oncogene suppressor protein. This syndrome is often underdiagnosed. Presence of lung cysts on chest CT should prompt inclusion of BHDS in the differential diagnosis, since these findings may develop earlier than other manifestations. There are key imaging characteristics of pulmonary cysts on CT of the chest which can suggest the diagnosis of BHDS and help in early detection and prompt screening for renal tumors. The main concern with BHDS is the increased risk of renal carcinoma. Here, we report a case of a 59-year-old male who was suspected to have the diagnosis of BHDS based on characteristic features of lung cysts on the Chest CT, subsequently confirmed by genetic testing.

## Background

Birt-Hogg-Dubé syndrome (BHDS) is a multiorgan systemic disorder manifesting as cutaneous fibrofolliculomas, pulmonary cysts with or without spontaneous pneumothorax, and renal tumors [1], [2], [3], [4].

The incidence of BHDS is unclear and it is often underdiagnosed [5]. It has an autosomal dominant inheritance with deletion mutation in the folliculin (FLCN) gene located on the short arm of chromosome 17 (17p12q11.2) [5]. The most characteristic finding of this syndrome is cutaneous Fibrofolliculoma. Fibrofolliculoma is a benign tumor of the hair follicle [5]. Pulmonary cysts are present in 80% to 100% of patients with this syndrome [3,4]. There is a high association with a risk of spontaneous pneumothorax, 32-fold higher compared to the general population [3,4]. Spontaneous pneumothorax occurs in about 24% to 38% of patients with BHDS [3,^6^,7]. Presence of pulmonary cyst and spontaneous pneumothorax are most common phenotypic manifestations of this syndrome, with extremely high rate of recurrence for pneumothorax. Pleurodesis is recommended following the initial episode of spontaneous pneumothorax to reduce future events [8].

The risk of renal tumor is higher in patients with BHDS, and renal cancer is the most threatening complication. The prevalence of renal tumors has been reported ranging from 6.5% to 34% [6,9]. Most renal tumors in patients with BHDS are malignant. Chromophobe renal cancer and a mixed pattern of chromophobe and oncocytic renal tumors are the most commonly reported histology. When these types of renal tumors are diagnosed, especially if multifocal or bilateral, evaluation for BHDS should be done [1].

The morphology and distribution of pulmonary cysts, as well as the clinical presentation, may be helpful in the differential diagnosis of BHDS. Lung cysts in BHDS syndrome occur bilaterally with lower and medial lung predominance, and involve costophrenic sulci. The cysts are scattered, multiple, well-circumscribed and thin-walled, with variable size. The size of lung cysts ranges from a few millimeters to 2 cm or more. The majority of the cysts are small (< 1 cm), with coexisting infrequent large lung cysts (> 2 cm). Pulmonary cysts of BHDS are usually oval, round, lenticular, or irregular in shape. Large cysts when present are multiseptated and irregular in shape, and usually located in the lung bases. The surrounding lung parenchyma is usually normal. Subpleural and fissural cysts are other common CT findings in BHDS. The cysts may abut or include the proximal lower pulmonary vessels. The number and size of the cysts in BHDS do not progress over time, unlike other diffuse cystic lung diseases [1,^10^,11].

Here, we report the characteristic lung findings in patients with Birt-Hogg-Dubé with a brief review of the literature.

## Case report

A 59-year-old male with past medical history of hypothyroidism, atrial fibrillation and atopic rhinitis presents to our tertiary care center for follow-up CT chest for incidentally detected pulmonary cysts on CT Chest Abdomen and Pelvis as part of a trauma workup. He was afebrile and had stable vitals. No history of smoking, dyspnea, chest pain, cough, fatigue or fever.

**Fig. 1:**
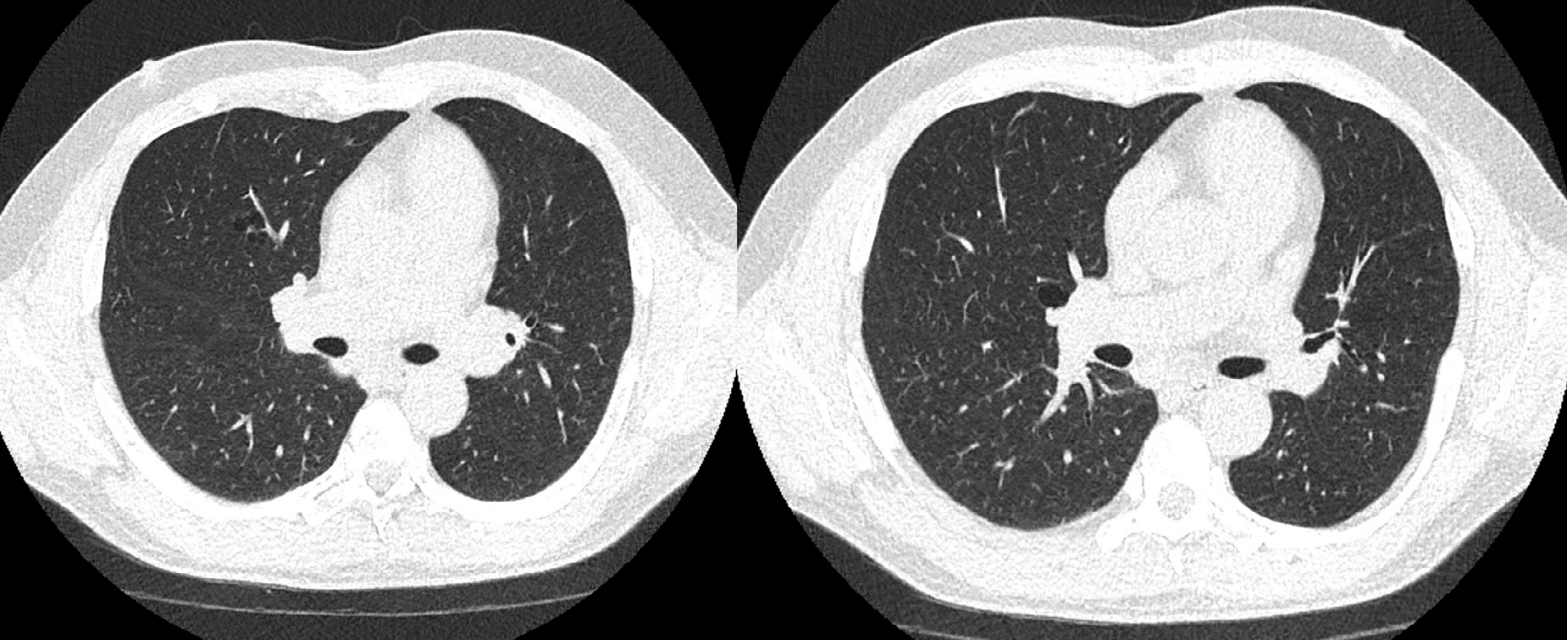
CT Chest without contrast: Axial images demonstrate multiple thin-walled elliptical well-defined para-mediastinal cysts in a basilar distribution with preserved lung volume and no evidence of interstitial lung disease

**Fig. 2:**
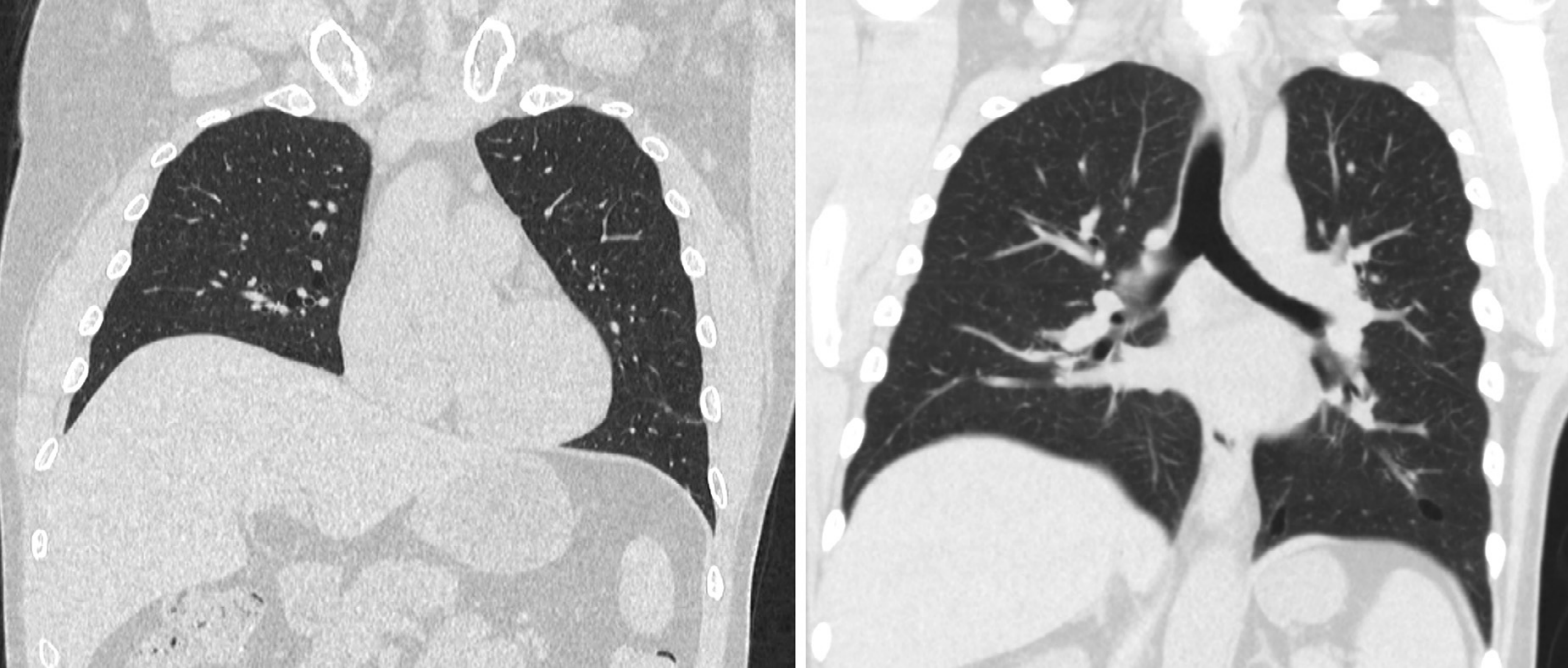
CT Chest without contrast: Coronal images demonstrate multiple thin-walled elliptical well-defined para-mediastinal air-filled cysts in a basilar distribution with preserved lung volume and no evidence of interstitial lung disease

**Fig. 3:**
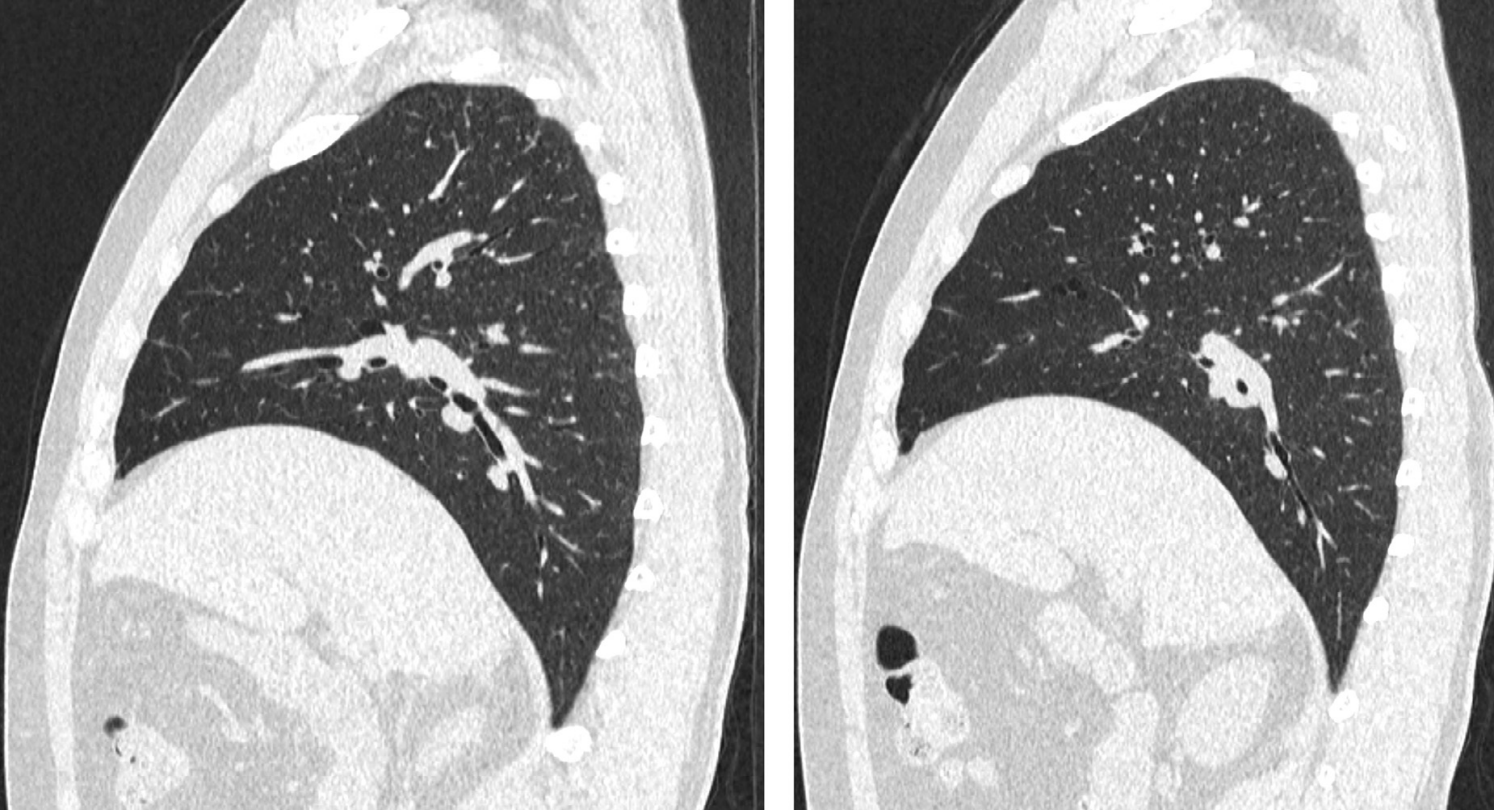
CT Chest without contrast: Sagittal images demonstrate multiple thin-walled elliptical well-defined para-mediastinal air-filled cysts in a basilar distribution with preserved lung volume without evidence of interstitial lung disease

CT Chest (Fig. 1, Fig. 2, Fig. 3) was performed which demonstrated multiple thin elliptical well-defined para-mediastinal air-filled cysts without internal structure, in a basilar distribution with preserved lung volume and no evidence of interstitial lung disease. The distribution pattern was concerning for cystic lung disease associated with Birt-Hogg-Dubé syndrome. The patient had no personal or family history of thyroid cancer, renal cancer or spontaneous pneumothorax.

He subsequently underwent genetic testing. Analysis of 106 gene revealed a pathogenic variant in the FLCN gene, FLCN c.1285dup (p.His429Profs*27) heterozygous, confirming the diagnosis of Birt-Hogg-Dubé syndrome.

## Discussion

Birt-Hogg-Dubé syndrome (BHDS) is named after Canadian physicians Arthur R Birt, Georgina R Hogg and W James Dubé who first reported this syndrome in 1977 [2]. BHDS is a rare condition, often underdiagnosed. Most cases are incidentally detected on CT or during workup of spontaneous pneumothoraces. There is no gender predisposition; men and women are equally affected. Patients often have a family history of recurrent pneumothoraces [1,2].

According to European Birt-Hogg-Dubé consortium guidelines [5] for the diagnosis of BHDS, one major or two minor criteria are required for the diagnosis. Major criteria include: (1) Five or more fibrofolliculomas with at least one confirmed histologically, and (2) Identification of a heterozygous pathogenic variant in FLCN. Minor criteria include: (1) Multiple pulmonary cysts (bilateral basal predominance) with no other apparent cause, with or without spontaneous pneumothorax, (2) Early-onset renal cancer (age <50 years), (3) Multifocal or bilateral renal cancer, (4) Renal cancer of mixed chromophobe and oncocytic histology, and (4) First-degree relative with BHDS [5].

Sasso et al [12] proposed stratifying pulmonary manifestations of BHDS into three categories: Definite pulmonary BHDS, Probable pulmonary BHDS and Possible pulmonary BHDS. (1) Definite pulmonary BHDS: Characteristic or compatible lung findings on high-resolution CT (HRCT) and skin biopsy positive for fibrofolliculoma; Characteristic or compatible HRCT and confirmed family history of BHDS in first- or second-degree family member; Characteristic or compatible HRCT and tissue confirmation of renal chromophobe adenoma or oncocytoma; Characteristic or compatible HRCT and tissue confirmation of genetic testing positive for BHDS. (2) Probable pulmonary BHDS: Characteristic HRCT, exclusion of Tuberous Sclerosis Complex (TSC) and Lymphangioleiomyomatosis (LAM), and personal or family history of pneumothorax; Compatible HRCT, exclusion of TSC and LAM, and any of the following: Family or personal history of renal tumors; Skin angiofibroma; and Renal angiomyolipoma. 3) Possible pulmonary BHDS: Compatible or characteristic HRCT.

Sasso et al [12] further defined lung HRCT findings as: Characteristic lung HRCT findings and Compatible HRCT findings. (1) Characteristic lung HRCT findings: Multiple thin-walled round, elliptical or lentiform well-defined air-filled cysts, without internal structure, in a basilar, medial and subpleural predominant distribution, with preserved or increased lung volume, and no other significant pulmonary involvement (specifically no interstitial lung disease). (2) Compatible HRCT findings: Thin-walled cysts without the more typical elliptical shape or subpleural distribution.

BHDS has an autosomal dominant inheritance with deletion mutation in the folliculin (FLCN) gene (17p11.2). There are about 142 unique DNA mutations of the FLCN gene which have been implicated with BHDS, which results in variable presentations. FLCN exact function is unknown but is thought to be an oncogene suppressor protein which may affect proteolytic metalloproteinase enzymes leading to lung matrix breakdown, tissue destruction and cyst formation [2].

The risk of renal tumor is higher in patients with BHDS, and renal cancer is the most threatening complication. Hence, early detection and screening for renal cancer is the key for improved survival. Patients have higher-risk for renal tumor, both benign and malignant. They are at increased risk of non-frequent renal cancers, such as chromophobe oncocytomas (50%), and chromophobe carcinomas (34%). Frequent renal cancers such as clear cell carcinomas (9%), oncocytomas (5%), and papillary renal cell cancers (2%) are also seen, but less frequently compared to the general population [2,12].

Management depends on the presentation. Temporary improvement of folliculomas is achieved through surgical and laser treatment. Pneumothoraces are managed with pleurodesis for recurrence prevention. Renal tumors are treated with nephron-sparing surgery when possible. Preventative recommendations include: Full-body skin examination every six to twelve months for potential risk of melanoma. Annual MRI or ultrasound to screen for renal lesion. It is strongly recommended to avoid use of cigarette smoking, high ambient pressures, and radiation exposure. First-degree relative should undergo molecular genetic testing for the family-specific pathogenic variant [3,^6^,^7^,13].

Birt-Hogg-Dubé syndrome is a rare yet important genetic condition that remains underdiagnosed partly due to variable clinical presentations. The presence of multiple skin lesions, renal cysts / lesions, and/or family history of pneumothorax are important diagnostic clues. Pulmonary cysts on imaging studies may be the initial manifestation and are often the finding to first suggest the diagnosis, highlighting the role of CT in early diagnosis. Awareness of the clinical manifestation of BHDS by radiologists is pivotal in early detection and management of complications, particularly renal malignancy [12].
